# Determinants of hospital and one-year mortality among older patients admitted to intensive care units: results from the multicentric SENIOREA cohort

**DOI:** 10.1186/s13613-021-00804-w

**Published:** 2021-02-17

**Authors:** Julien Demiselle, Guillaume Duval, Jean-François Hamel, Anne Renault, Laetitia Bodet-Contentin, Laurent Martin-Lefèvre, Dominique Vivier, Daniel Villers, Montaine Lefèvre, René Robert, Philippe Markowicz, Sylvain Lavoué, Anne Courte, Eddy Lebas, Stéphanie Chevalier, Cédric Annweiler, Nicolas Lerolle

**Affiliations:** 1 Service de Médecine Intensive Réanimation et Médecine Hyperbare, Centre Hospitalier Universitaire, 4, Rue Larrey, 49933 Angers Cedex 9, France; 2 Department of Geriatric Medicine, Angers University Hospital, 4 rue du Larrey, 49933 cedex 9 Angers, France; 3 Angers University Memory Clinic, Research Center on Autonomy and Longevity, UPRES EA 4638, University of Angers, 4 rue du Larrey, 49933 cedex 9 Angers, France; 4 Maison de la Recherche, Centre Hospitalier Universitaire, 4, Rue Larrey, 49933 Angers Cedex 9, France; 5 Service de Réanimation Médicale, Centre Hospitalier Universitaire, Boulevard Tanguy Prigent, 29609 Brest, France; 6 Hôpital Bretonneau, Service de Réanimation Médicale, Centre Hospitalier Régional Universitaire de Tours, 2 Bis Boulevard Tonnellé, 37044 Tours Cedex 09, France; 7 Service de Réanimation Polyvalente, Centre Hospitalier Départemental Vendée-Hôpital de La-Roche-sur-Yon, Les Oudairies, 85925 La-Roche-sur-Yon Cedex 09, France; 8 Service de Réanimation Médico-Chirurgicale, Centre Hospitalier du Mans, 194 Avenue Rubillard, 72037 Le Mans Cedex 09, France; 9 Hôtel-Dieu, Service de Médecine Intensive et Réanimation, Centre Hospitalier Universitaire de Nantes, 30 bd Jean Monnet, 44093 Nantes, France; 10 Centre Hospitalier Des Pays de Morlaix, Service de Réanimation Polyvalente, 15, Rue de Kersaint Gilly, BP 97237, 29672 Morlaix Cedex, France; 11 CHU de Poitiers, Service de Réanimation Médicale, 2, Rue de la Milétrie, CS 90577, 86021 Poitiers Cedex, France; 12 Centre Hospitalier de Cholet, Service de Réanimation Polyvalente, 1 Rue de Marengo, BP 507, 49325 Cholet Cedex, France; 13 Centre Hospitalier Universitaire de Rennes, Hôpital Pontchaillou, Unité de Réanimation Médicale, 2, Rue Henri Le Guilloux, 35033 Rennes Cedex 9, France; 14 Centre Hospitalier de Saint Brieuc, Service de Réanimation Polyvalente, 10, Rue Marcel Proust, BP 2367, 22027 Saint Brieux Cedex 01, France; 15 Centre Hospitalier Bretagne Atlantique, 20 Boulevard Général Maurice Guillaudot, BP 70555, 56017 Vannes Cedex, France; 16 Centre Hospitalier de Saint Malo, Service de Réanimation Polyvalente, 1, Rue de la Marne, 35403 Saint Malo Cedex, France; 17 Robarts Research Institute, Department of Medical Biophysics, Schulich School of Medicine and Dentistry, The University of Western Ontario, London, ON Canada

**Keywords:** Critical care outcomes, Older adults, Outcome assessment (health care), Mechanical ventilation

## Abstract

**Background:**

Improving outcomes of older patients admitted into intensive care units (ICU) is a raising concern. This study aimed at determining which geriatric and ICU parameters were associated with in-hospital and long-term mortality in this population.

**Methods:**

We conducted a prospective multicentric observational cohort study, including patients aged 75 years and older requiring mechanical ventilation, admitted between September 2012 and December 2013 into ICU of 13 French hospitals. Comprehensive geriatric assessment at ICU admission and ICU usual parameters were registered in a standardized manner. Survival was recorded and comprehensive geriatric assessment was updated after 1 year during a dedicated home visit.

**Results:**

501 patients were analyzed. 108 patients (21.6%) died during the hospital stay. One-year survival rate was 53.8% (IC 95% [49.2%; 58.2%]). Factors associated with increased in-hospital mortality were higher acute illness severity score, resuscitated cardiac arrest as primary ICU diagnosis, perception of anxiety and low quality of life by the proxy, and living in a chronic care facility before ICU admission. Among patients alive at hospital discharge, factors associated with increased 1-year mortality in multivariate analysis were longer duration of mechanical ventilation, all primary ICU diagnoses other than septic shock, a Katz-activities of daily living (ADL) score below 5 and living in a chronic care facility before ICU admission. Among the 163 survivors at 1 year who received a second comprehensive geriatric assessment, the ADL score (functional abilities) showed a significant but moderate decline over time, whereas the Mini-Zarit score (family burden) improved. No significant change in patients’ place of life was observed after 1 year, and quality of life was reported as happy-to-very-happy in 88% of survivors.

**Conclusions:**

The mortality rate remains high among older ICU patients requiring mechanical ventilation. Factors associated with short- and long-term mortality combined geriatric and ICU criteria, which should be jointly evaluated in routine care.

*Clinical trial registration* NCT01679171

## Background

Admission of elderly patients represents around 15% of all patients admitted into intensive care units (ICU) [1, 2]. Efforts to improve older patient care and outcomes thus become a major concern for intensivists. These patients raise the problem of their profile and resilience which can vary considerably, due to a wide range of comorbidities and acute diseases [3, 4]. Such variance impacts both the short- and long-term outcomes [5]. Moreover, most recent interventional trials in the ICU failed to show any relevant effects, justifying a call for more personalized care, which may be all the more relevant in older patients [6]. Indeed, taking into account all the features of older patients may help to provide the most appropriate care from the decision to ICU admission, ICU organ support initiation and post-ICU care. We hypothesized that the prognosis of older patients admitted into ICU may depend on both geriatric and ICU parameters, and that these parameters may combine differently for predicting short- and long-term outcomes.

In this multicentric longitudinal prospective observational ICU-based cohort study, we aimed at determining, among a wide array of geriatric and ICU characteristics at admission, which factors were associated with short- and long-term mortality (in-hospital and 1-year mortality) in patients aged 75 years and older admitted into ICU and requiring mechanical ventilation. In addition, we aimed at refining the 1-year outcomes beyond survival using patients’ evaluation on the place of life.

## Methods

### Participants

We studied participants of the SENIOREA cohort study, a French large observational prospective multicentric cohort study designed to better understand the contribution of ICU in older patients in terms of long-term survival and quality of life. From September 2012 to December 2013, all consecutive subjects aged 75 years and older, requiring initiation of mechanical ventilation (either invasive or non-invasive) over the 48 h following admission, were recruited in 13 ICUs in Western France. Follow-up included patients and the most relevant proxy for each included patient when available. The Angers People Protection Committee (Comité de Protection des Personnes Ouest II) approved the study (No. 2012/09). Requirement for mechanical ventilation was retained as a marker of acute severity and being the most frequent organ support justifying ICU admission [2]. Non-French-speaking patients were excluded as well as patients living outside Western part of France, and patients already included in the study. According to patients’ preference when possible, one proxy was selected as the “corresponding proxy”. Patients and corresponding proxies received oral and written information and written consent was obtained for both before inclusion. In case of a patient unable to consent, the proxy gave consent for him/herself and for the patient’ participations. Written consent of the patient was obtained as soon as deemed possible considering the patient status. In the absence of any proxy, patients could be included only if they could consent by themselves at ICU admission.

### ICU measures

Baseline demographic characteristics were registered and all patients underwent an admission comprehensive geriatric assessment through interview of the proxy and review of available medical files in the 48 h following ICU admission. This standardized assessment covered 13 health components: comorbidity assessed with the Cumulative Illness Rating Scale for Geriatrics score (CIRS-G) [7], frailty (assessed using the Study of Osteoporotic Fracture (SOF) Criteria for Frailty, a score of 0 suggests robust, of 1 suggests prefrail, and higher or equal to 2 suggests frailty [8]), family burden (Mini-Zarit score [9]), activities of daily living (ADL) score [10], Instrumental Activities of Daily Living (IADL) score [11], nutritional status (body mass index), and perceived quality of life assessed through the single last question from the Perceived quality of life scale “To your opinion, how happy was the patient?” with a 5-point response scale from extremely unhappy to very happy [12]. History of falls (number of falls occurrence in the previous year), cognitive disorders, anxiety and depression were assessed through closed questions (for example: “To your opinion, was the patient anxious most of the time: yes/no?”) (see Additional file 1). Proxies were interrogated on their estimation of the best status in the 3 preceding months. In the absence of a proxy, the patient, the corresponding physician and all implicated caregivers were interviewed. On the first 24 h of ICU admission, acute severity parameters allowing to calculate SOFA (Sepsis-related Organ Failure Assessment) and SAPS II (Simplified Acute Physiology Score II) scores [13, 14], and the main diagnosis (selected in a pre-defined list of ICU diagnosis) were collected. At the end of hospital stay, duration of organ support, hospital length of stay, withdrawal of care decision, site of discharge and in-hospital mortality were collected.

### One-year follow-up

At 1 year after ICU admission, deaths were ascertained from an informant during a telephone interview and/or by reviewing obituaries with a completion rate of 100%. In survivors, a home-based comprehensive geriatric assessment was proposed to the patient and the corresponding proxy at 1 year after hospital discharge. Upon agreement of the patients, a trained research assistant arranged an on-site evaluation of the patient and the corresponding proxy directly in his/her living space. The home-based comprehensive geriatric assessment explored the same 13 health components as previously cited, using validated scores: depression through the Geriatric Depression Scale-4 items (4-item GDS, a score equal or higher than 2/4 suggests depressive state) [15], anxiety using the Covi Anxiety Scale (a score equal or higher than 6/15 is suggestive for anxiety) [16], mobility with the Five Times Sit-to-Stand test [17], cognition with the Mini-Mental State Examination (a score under 25 was considered for the diagnosis of cognitive disorder) [18], and pain with the Verbal Rating Scale [19]. The proxy was specifically interrogated on the family burden (Mini-Zarit score, a score equal or higher than 2/7 is suggestive for a heavy family burden [9]). Such a home visit conducted by a research assistant after a hospitalization in ICU had already demonstrated its feasibility and ability to collect contributory information in the pilot pre-SENIOREA study [20].

### Statistical analyses

Categorical data were presented as percentages and compared using Fisher exact tests while the groups compared were independent, or using tests of symmetry for comparisons of paired data. Continuous data were presented as mean ± standard deviation and compared using Kruskal–Wallis rank tests for independent data or Friedman tests for paired data.

To identify factors associated with hospital mortality and 1-year mortality as hazard ratios (HRs), Cox regression models were computed. For the factors associated with 1-year mortality, only patients who were discharged alive from the hospitalization were included in the analysis. The assumption of proportional hazards was tested by analyzing Schoenfeld residuals [21]. In the first step, univariate analyses were conducted for every inclusion characteristics variable independently of each other. In the second step, multivariate Cox regression models were built using variables with *p* value < 0.2 in univariate analysis. When covariates were strongly correlated, only one was kept in the multivariate model, based on clinical considerations (for example, SOFA and IGS-II could not both be included in the model, only IGS-II was kept). Continuous variables were kept as continuous for descriptive purpose in univariate analyses, but only categorized data were kept in multivariate analysis (for example IADL, divided in IADL under 4 and equal or above 4).

All tests were two sided, with a type I error set at 0.05. Analyses were performed using Stata 14.2 (StataCorp LP, College Station, TX).

## Results

**Fig. 1:**
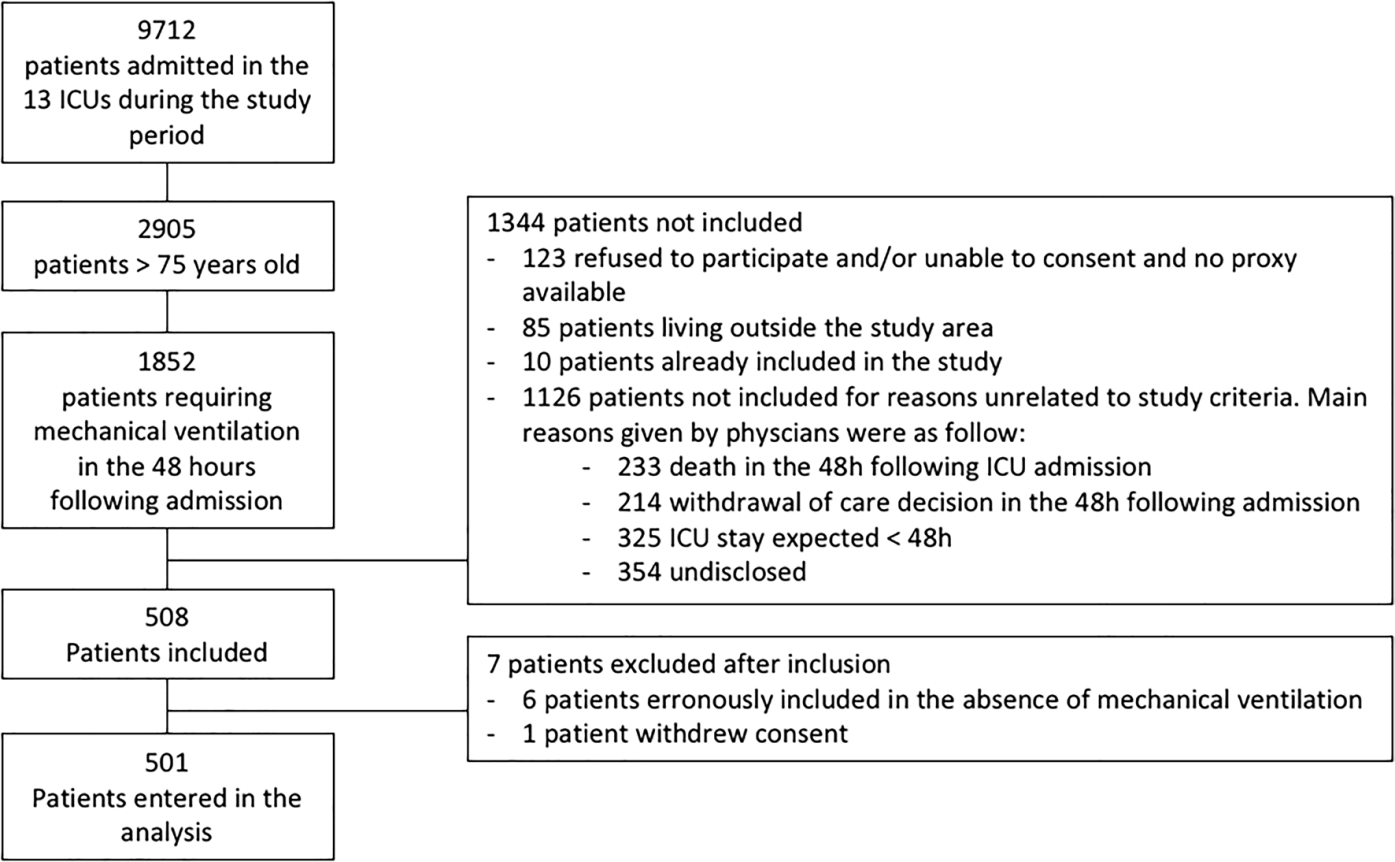
Flowchart of the study

Five hundred and eight patients were initially included in the study, 6 patients were secondarily excluded due to erroneous inclusion in the absence of mechanical ventilation and one patient withdrew his consent at the end of the ICU stay. Thus 501 patients were finally entered in the analysis (see Fig. 1 for flowchart of the study).

Baseline characteristics, admission comprehensive geriatric assessment and ICU/hospital data are presented in Tables 1 and 2, respectively. Patients included in the SENIOREA study were old (mean age 80.4 ± 4.1 years), seriously ill (admission SAPS II 54 ± 17) with a need for mechanical ventilation (invasive or not) in all patients, in accordance with the inclusion criteria. More than 90% of patients lived at home, with disability (ADL < 5) in 22%.

**Table 1:** Baseline characteristics and admission comprehensive geriatric assessment of the 501 included patients

	Number of patients with data available	Patients included (*n* = 501)
Age (years)	501	80.4 ± 4.1
Sex (male)	501	268 (53%)
Residential living^a^	501	
Chronic care facility		5 (1%)
Supervised residence setting		25 (5%)
At home, alone		156 (31%)
At home, with someone else		305 (61%)
Other		10 (2%)
Legal protection	498	
Yes		13 (3%)
No		485 (97%)
Presence of a caring proxy	499	
Yes		385 (77%)
None		114 (23%)
Presence of a corresponding proxy	501	
Yes		468 (93%)
None		33 (7%)
Caring professional at home on a regular basis^b^	495	
Yes		126 (26%)
None		369 (74%)
Admission comprehensive geriatric assessment		
Cumulative Illness Rating Scale for Geriatrics^c^ (/60)	501	8.3 ± 4.3
Frailty: SOF criteria	416	
Robust		142 (34%)
Prefrail		155 (37%)
Frail		119 (29%)
Family burden: Mini-Zarit Score (/7)	468	1.5 ± 1.41
Cognition impairment: yes	469	139 (30%)
Presence of depressive symptoms: yes	469	170 (36%)
Geriatric Depression Score 4-items score	296	1.6 ± 0.91
Geriatric Depression Score 4-items ≥ 2	296	127 (43%)
Presence of anxiety: yes	466	281 (60%)
Impaired mobility: yes	494	98 (20%)
Presence of chronic pain moderate to severe: yes	466	209 (45%)
Activity of daily living (ADL) (/6)	485	5.2 ± 1.3
Number of patients with score < 5	485	102 (21%)
Instrumental Activity of Daily Living (iADL) (/5)	480	3.0 ± 1.2
Number of patients with score < 4	480	257 (53.5%)
History of fall: yes	477	245 (51%)
Body max index (kg/m^2^)	471	28.5 ± 6.5
< 21		45 (9.6%)
[21–25]		101 (21.4%)
[25–30]		160 (34.0%)
≥ 30		165 (35.0%)
Perceived quality of life	464	
Very happy		74 (16%)
Happy		289 (62%)
Unhappy		89 (19%)
Very unhappy		12 (3%)

Data are *n* (% of patient with data available) or mean ± standard deviation

^a^In the 3 months preceding admission

^b^At least once weekly

^c^Higher score indicating higher number of comorbidities

**Table 2:** ICU characteristics of the 501 patients included in the SENIOREA study

	Number of patients with data available	Patients included (*n* = 501)
Location of the patient before ICU admission	501	
Acute facility care		290 (58%)
Home		172 (34%)
Chronic facility care		11 (2%)
Supervised residence setting		8 (3%)
Other		20 (4%)
Primary ICU diagnosis	498	
Septic shock		78 (15.7%)
Non-septic shock		22 (4.4%)
Resuscitated cardiac arrest		52 (10.4%)
Acute on chronic respiratory failure		64 (12.9%)
Acute respiratory failure		149 (29.9%)
Acute kidney injury and metabolic disorders		11 (2.2%)
Neurological disorder		51 (10.2%)
Post-surgery		26 (5.2%)
Drug overdose		5 (1%)
Trauma		7 (1.4%)
Other		33 (6.6%)
Admission SOFA, *m* ± sd	501	7.0 ± 3.7
Admission SAPSII, *m* ± sd	501	54 ± 17
Type of mechanical ventilation, *n* (%)	489	
Invasive only		290 (59.3%)
Non-invasive only		98 (20.0%)
Combined		101 (20.7%)
Total length of mechanical ventilation (days), *m* ± sd	463	7.5 ± 10.0
Med (IQR)		4 (2–10)
Renal replacement therapy requirement	498	70 (14.1%)
Total length of renal replacement therapy, *m* ± sd	70	7.6 ± 9.3
Med (IQR)		4 (1–11)
Vasopressor requirement	497	252 (50.7%)
Total length of vasopressor infusion (days), *m* ± sd	251	4.4 ± 6.2
Med (IQR)		2 (1–5)
ICU length of stay (days), *m* ± sd	499	10.5 ± 13.8
Med (IQR)		2 (1–5)
Hospital length of stay (days), *m* ± sd	499	20.2 ± 23.3
Med (IQR)		14 (7–26)
ICU mortality	499	108 (21.6%)
Hospital mortality	499	130 (26.0%)
Limitation of life sustaining therapies, *n *(%)	447	135 (30.2%)

Data are *n* (%), mean (*m*) ± standard deviation (sd) or median (med) with interquartile range (IQR). ICU, intensive care unit, SAPS, Simplified Acute Physiology Score, SOFA, Sequential Organ Failure Assessment

**Fig. 2:**
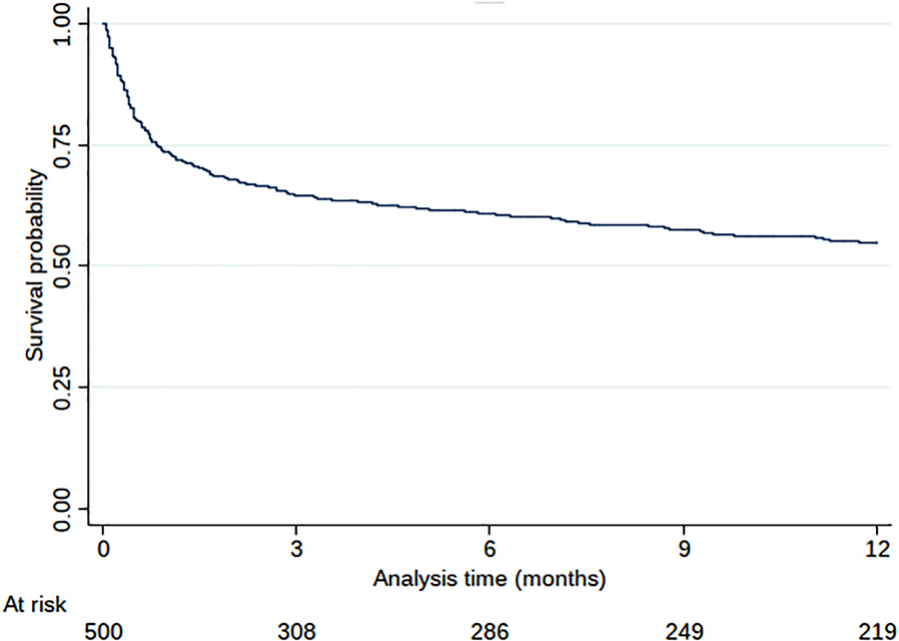
Kaplan–Meier survival curve of the patients included in the SENIOREA study

The 1-year survival rate for the 501 patients initially admitted in the ICU was 53.8% (IC 95% [49.2%; 58.2%]). Kaplan–Meier curve is presented in Fig. 2. One hundred and eight patients (21.6%) died during the ICU stay. Limitation of life sustaining therapies was decided in 135 patients, in whom 89 died during the hospitalization. Among the sub-sample of the 371 patients discharged alive from the hospital, one-year survival rate was 72.8% (IC 95% [68.2%; 77.3%]).

Among the 270 survivors at 1 year, the “at home” comprehensive geriatric assessment could be completed in 163 patients (60%). Table 3 shows baseline and one-year data of these patients. The ADL score showed significant but moderate decline over time, whereas Mini-Zarit score (family burden) improved at 1 year. Of note, there was no significant change in patients site of living after 1 year, and quality of life was reported as happy-to-very-happy in 88% of survivors. By contrast 84% of patients had a Geriatric Depression Scale ≥ 2, indicating depressive symptoms. Data showed a low frequency of pain, anxiety and cognitive impairment.

**Table 3:** Characteristics of survivors after 1 year of follow-up

	ICU admission	one-year evaluation	*p*-value
IADL score (/8) (*n* = 109)	3.26 ± 0.99	2.96 ± 1.35	0.05
< 4	65 (44.8%)	52 (47.7%)	0.84
≥ 4	80 (55.2%)	57 (52.3%)	
ADL score (/6) (*n* = 109)	5.55 ± 0.98	5.24 ± 1.41	0.06
< 5	16 (11%)	24 (22%)	< 0.01
≥ 5	130 (89%)	85 (78%)	
Cognitive impairment (MMSE, /30) (*n* = 92)			
Yes (< 25)		11 (12%)	£
No		81 (88%)	
Anxiety (COVI test, /12) (*n* = 135)			
Yes (> 6)		2 (1.5%)	£
No		133 (98.5%)	
Geriatric Depression Scale-4 items (*n* = 132)			
Mean			£
< 2		21 (16%)	
≥ 2		111 (84%)	
Pain (*n* = 138)			
No		55 (39.9%)	£
Mild		22 (15.9%)	
Moderate		47 (34.1%)	
Severe		14 (10%)	
Fall (*n* = 109)			
Yes		37 (33.9%)	£
No		70 (64.2%)	
Unknown		2 (1.83%)	
Five Times Sit-to-Stand Test, seconds (*n* = 51)		21.3 ± 7.9	£
QoL—happiness (*n* = 138)			£
Very happy		28 (20.3%)	
Happy		93 (67.4%)	
Unhappy		17 (12.3%)	
Very unhappy		0 (0%)	
Mini-Zarit score (/7) (*n* = 101)	1.28 ± 1.17	0.82 ± 1.18	< 0.001
< 2	96 (72.7%)	85 (84.2%)	
≥ 2	36 (27.3%)	16 (15.8%)	0.11
Residential living (*n* = 146)			
Home	142 (94%)	134(92%)	
Chronic facility care/supervised residence setting	9 (6%)	12 (8%)	0.39
Living alone (*n* = 144)			
Yes	109 (72.7%)	102 (70.8%)	0.65
No	41 (27.3%)	42 (29.2%)	

Data are *n* (%) or mean ± standard deviation. The number in brackets *n* refers to the number of patients with available data among the 163 patients evaluated at 1 year

ADL, Activities of Daily Living, GDS, Geriatric Depression Scale, IADL, Instrumental Activities of Daily Living, MMSE, Mini-Mental State Evaluation, QoL, Quality of Life

£ Admission data and comparisons are not shown as the data were collected using different tools at admission (proxy interrogation, typically through yes/no questions) and at one year (direct patient evaluation using standardized questionnaires)

In multivariate analysis, factors associated with increased in-hospital mortality (see Table 4 for univariate and multivariate analyses) were higher acute illness severity score, resuscitated cardiac arrest as primary ICU diagnosis, perception of anxiety and of low quality of life by the proxy, living in a chronic care facility before ICU admission. Among patients alive at hospital discharge, factors associated with increased one-year mortality (see Table 5) in multivariate analysis, were longer duration of mechanical ventilation, all primary ICU diagnoses other than septic shock, a Katz-ADL below 5 and living in a chronic care facility before ICU admission.

**Table 4:** Determinants associated with in-hospital mortality using Cox cause-specific model

	Univariate			Multivariate		
	HR	95% CI	*p*	HR	95% CI	*p*
Gender (reference: male)	0.76	0.5–1.14	0.178	–	–	–
Admission SOFA Score (per 1 pt increment)	1.12	1.06–1.18	< 0.001	–	–	–
Admission SAPS2 Score (per 1 pt increment)	1.03	1.02–1.04	< 0.001	1.02	1.00–1.03	0.044
Primary ICU diagnosis (reference septic shock)						
Non-septic shock	1.01	0.38–2.64	0.989	0.51	0.16–1.56	0.237
Resuscitated cardiac arrest	2.71	1.57–4.68	< 0.001	2.29	1.17–4.48	0.015
Acute on chronic respiratory failure	0.58	0.26–1.30	0.187	0.95	0.36–2.53	0.921
Acute respiratory failure	0.46	0.24–0.87	0.017	0.55	0.25–1.21	0.139
Neurological disorder	0.96	0.51–1.83	0.912	1.14	0.49–2.64	0.758
Post-surgery	0.25	0.06–1.04	0.057	0.34	0.07–1.57	0.168
Other	0.56	0.23–1.36	0.200	0.75	0.26–2.12	0.582
Invasive mechanical ventilation	2.56	1.11–5.89	0.027	1.48	0.52–4.21	0.466
RRT requirement	1.52	1.00–2.31	0.051	1.53	0.86–2.71	0.147
Vasopressor requirement	1.34	0.87–2.08	0.188	1.07	0.60–1.90	0.814
CIRS for geriatrics (per 1 pt increment)	0.99	0.94–1.03	0.560	–	–	–
CIRS for geriatrics < 8	0.76	0.52–1.12	0.171	–	–	–
Residential living (reference: chronic care facility)						
Supervised residence setting	0.25	0.05–1.11	0.068	0.15	0.03–0.84	*0.030*
At home	0.25	0.08–0.82	0.021	0.17	0.05–0.62	*0.007*
IADL (per 1 pt increment)	0.99	0.84–1.17	0.905	–	–	–
IADL ≥ 4	0.99	0.68–1.47	0.998	–	–	–
Katz-ADL (per 1 pt increment)	0.97	0.83–1.13	0.705	–	–	–
Katz-ADL ≥ 5	0.88	0.54–1.42	0.598	–	–	–
History of fall (reference: no)						
Yes	1.05	0.71–1.56	0.789	–	–	–
Unknown	0.83	0.11–6.0	0.855	–	–	–
Cognitive impairment	0.96	0.63–1.48	0.872	–	–	–
Perceived quality of life (unhappy, very unhappy)	1.85	1.20–2.87	0.006	1.95	1.12–3.41	*0.018*
Chronic pain (reference: no)						
Mild	1.08	0.62–1.89	0.781	–	–	–
Moderate	1.31	0.79–2.18	0.295	–	–	–
Severe	1.01	0.57–1.77	0.987	–	–	–
Presence of anxiety: yes	1.36	0.90–2.05	0.140	1.64	1.00–2.69	0.051
Presence of depressive symptoms	1.17	0.79–1.74	0.424	–	–	–
Mini-Zarit (per 1 pt increment)	1.03	0.91–1.17	0.641	–	–	–
< 2	1.00	0.65–1.52	0.990	–	–	–
Frailty (reference: robust)						
Prefrail	1.69	1.01–2.82	0.045	1.59	0.90–2.80	0.112
Frail	1.59	0.93–2.71	0.092	1.03	0.50–2.14	0.933

ADL, Activity of daily living, CIRS for geriatrics, Cumulative Illness Rating Scale for Geriatrics, IADL, Instrumental Activity of Daily Living, ICU, intensive care unit, RRT, renal replacement therapy, SAPS, Simplified Acute Physiology Score, SOFA, Sequential Organ Failure Assessment

CIRS for geriatrics under 8 is suggestive of few comorbidities

**Table 5:** Determinants associated with one-year mortality among patients who survived to hospitalization using Cox cause-specific model

	Univariate			Multivariate		
	HR	95% CI	*p*	HR	95% CI	*p*
Gender (reference: male)	0.82	0.56–1.19	0.292	–	–	–
Admission SOFA Score (per 1 pt increment)	1.00	0.95–1.06	0.895	–	–	–
Admission SAPS2 Score (per 1 pt increment)	1.02	1.0–1.03	0.009	1.01	1.00–1.03	0.129
Primary ICU diagnosis (reference septic shock)						
Non-septic shock	2.82	1.05–7.58	0.040	4.60	1.12–18.79	0.034
Resuscitated cardiac arrest	2.18	0.84–5.66	0.108	5.82	1.63–20.83	0.007
Acute on chronic respiratory failure	1.90	0.85–4.23	0.116	3.85	1.09–13.60	0.037
Acute respiratory failure	1.40	0.67–2.92	0.377	3.46	1.08–11.14	0.037
Acute kidney injury/metabolic disorders	1.89	0.51–7.00	0.338	4.99	1.00–24.93	0.050
Neurological disorder	2.84	1.24–6.49	0.013	6.70	2.01–22.33	0.002
Post-surgery	1.82	0.68–4.88	0.236	5.80	1.39–24.25	0.016
Other	1.38	0.55–3.48	0.493	4.97	1.40–17.69	0.013
Invasive mechanical ventilation	1.23	0.79–1.92	0.351	–	–	–
Length of mechanical ≥ 3 days (reference < 3 days)	1.62	1.06–2.47	0.025	1.86	1.06–3.26	0.031
Length of RRT (reference no RRT)						
< 3 days	0.46	0.11–1.86	0.275	0.77	0.17–3.52	0.739
≥ 3 days	2.40	1.25–4.61	0.008	2.34	0.77–7.13	0.135
Length of vasopressor requirement (reference no vasopressor)						
< 3 days	0.89	0.56–1.43	0.640	–	–	–
≥ 3 days	1.26	0.78–2.03	0.352	–	–	–
CIRS for geriatrics (per 1 pt increment)	1.03	0.99–1.08	*0.146*	1.01	0.95–1.07	0.747
CIRS for geriatrics < 8	1.20	0.82–1.77	0.344	–	–	–
Residential living (reference chronic care facility)						
Supervised residence setting	0.20	0.02–1.58	0.126	0.09	0.01–0.86	0.036
At home	0.16	0.02–1.15	0.068	0.10	0.01–0.88	0.038
IADL (per 1 pt increment)	0.77	0.66–0.90	0.001	–	–	–
IADL ≥ 4	0.52	0.34–0.79	0.002	0.75	0.41–1.36	0.339
Katz-ADL (per 1 pt increment)	0.78	0.69–0.89	< 0.001	–	–	–
Katz-ADL ≥ 5	0.41	0.28–0.61	< 0.001	0.53	0.30–0.96	0.038
History of fall (reference: no)						
Yes	1.02	0.69–1.5	0.924	–	–	–
Unknown	2.19	0.30–15.83	0.438	–	–	–
Cognitive impairment	1.14	0.76–1.72	0.526	–	–	–
Perceived quality of life (unhappy, very unhappy)	1.59	1.03–2.45	0.036	1.11	0.62–1.98	0.723
Chronic pain (reference: no)						
Mild	0.89	0.50–1.59	0.699	–	–	–
Moderate	1.34	0.82–2.18	0.236	–	–	–
Severe	1.10	0.63–1.92	0.733	–	–	–
Presence of anxiety	1.17	0.79–1.75	0.431	–	–	–
Presence of depressive symptoms	1.32	0.89–1.96	0.164	0.90	0.52–1.55	0.698
Mini-Zarit (per 1 pt increment)	1.24	1.09–1.41	0.001	–	–	–
< 2	1.71	1.14–2.58	0.010	1.13	0.64–1.99	0.664
Frailty (reference: robust)						
Prefrail	1.28	0.74–2.22	0.377	0.96	0.50–1.85	0.903
Frail	2.41	1.42–4.06	0.001	1.51	0.75–3.05	0.244

ADL, activity of daily living, CIRS for geriatrics, Cumulative Illness Rating Scale for Geriatrics, IADL, Instrumental Activity of Daily Living, ICU, intensive care unit, RRT, renal replacement therapy, SAPS, Simplified Acute Physiology Score, SOFA, Sequential Organ Failure Assessment

CIRS for geriatrics under 8 is suggestive of few comorbidities

## Discussion

This French large multicentric prospective cohort study showed that among older patients admitted into ICU with a need for mechanical ventilation, survival rate at one year was 54% and quality of life in these survivors seemed preserved. Mortality was associated with combinations of ICU and geriatric parameters. These parameters differed slightly for short- and long-term mortality. Living in a chronic facility care and primary ICU diagnoses were associated with both short- and long-term vital outcomes, in-hospital mortality was rather related to acute severity on admission and perception by the proxy of low quality of life in the months preceding admission, whilst one-year survival was related to the duration of organ support during the ICU stay and to functional abilities before ICU admission.

The impact of various pre-morbid conditions—referred to as frailty, geriatric condition, pre-morbid functional status, or loss of independence, among others—on ICU and short-term mortality in older patients has already been the matter of some research [5, 22–25]. Only few studies have performed yet a standardized comprehensive geriatric assessment in older ICU patients [20, 25] with a long-term follow-up, which is a major strength of the present study. In the largest prospective observational study conducted so far to assess the factors associated with 1-month mortality, Guidet et al. reported in multivariate analysis that frailty (Clinical Frailty Scale), SOFA, diagnosis and age were prognostic factors [26]. We confirm and extend these data by showing that a similar combination of ICU and geriatric parameters also predicts long-term survival. The fact that not exactly the same parameters were associated with short- and long-term outcomes is interesting: functional abilities before admission may predict long-term resilience after an acute event, which may be hampered by a prolonged requirement for organ support. Frailty and/or low quality of life may rather impair short-term resistance and/or willingness to survive to acute disease, all the more that this disease is severe. The poor outcome after resuscitated cardiac arrest due to cerebral lesions explains its major short-term impact, on the contrary the possibility of a total cure of an infectious event contrasts with irreversible organ damage (acute or pre-existing) often associated with other acute diseases such as cardiogenic shock, COPD exacerbation, coma.

One-year survival (54%) was relatively high compared to other studies in older patients, even more that we selected patients requiring mechanical ventilation. Most previous studies disclosed a 30% one-year survival in very old patients (i.e., aged 80 and older), even though proportion of patients requiring ventilation was below 70% [27, 28]. Recent improvement in the prognosis of older patients has been shown in several studies, with one-year survival rate close to that observed in our study [22, 25, 29]. Obviously, care of older patients, from triage to ICU care, is evolving very rapidly and update data are permanently required. Beyond survival, much controversy has emerged as to whether a surviving older patient after ICU care is a real success considering the risk of altered trajectory of life after ICU care [30]. Most longitudinal studies yielded mixed results, showing either acceptable or poor long-term quality-of-life [25]. However, these previous investigations have been limited by single-center enrolment, small sample sizes, and use of non-validated functional outcome measures. The relative good one-year prognosis observed in our study is reassuring, and, if ADL was lower at 1 year, we observed conversely that family burden was lower. This may suggest a global improvement in the global home care. Although it is indubitable that long-term functional and quality of life outcomes should remain a major concern, our study shows that one-year survival can be considered as a good proxy for ICU “success” in older patients. The discrepancy between perceived quality of life (happy/very happy) and the presence of depressive symptoms is, however, noticeable. It is likely due to the difference between the “constructing self” and the “experiencing self”. Experiencing self refers to the everyday “objective” feelings, interrogated here by the specific geriatric depression scale assessing symptoms. Constructing self refers to the perception of the value of his own life, this is interrogated by the perceived quality of life. Our results confirm that perceived and experienced quality of life are two different items that may not align [31]. In older patients, although everyday life is hampered by multiple concerns (pain, restricted mobility, social isolation, etc.), life itself may be cherished.

This study suffers from several biases, some of which have been already addressed. The number of patients who were not included in the absence of exclusion criteria is not negligible, and may induce a selection bias. Physicians aware that the goal of the study was to observe long-term outcome, may have been reluctant to include patients that they felt very unlikely to survive. This potential bias may have led to overestimate survival. Several years elapsed between inclusion period and publication of this article, and the clinical picture may accordingly have changed.

Comprehensive geriatric assessment, based on an extensive collection of data is a strength of our study. However, it may be hardly feasible in routine practice, although collecting such data should be considered as good medical practice. Shorter dedicated standardized questionnaires could be used in this perspective. In this setting, we have chosen tools for this evaluation that could be used easily at bedside: for example, the perceived quality of life is simple, albeit not validated among older patients [32]. Elsewhere, the SOF was chosen as it is one of the feasible tool in the ICU, and because it exhibits very good diagnostic efficiency to predict falls, disability, fracture and mortality risks compared to more complex tools such as the consensual Fried score [8].

Finally, an important proportion of surviving patients could not be evaluated at home after 1 year. A similar bias was observed in previous studies, illustrating the difficulty of achieving the long-term follow-up of ICU patients [25].

## Conclusions

In this study, one-year survival in older patients admitted into ICU and requiring mechanical ventilation was 54%, with satisfactory functional outcomes. Living in a chronic care facility before ICU admission impacted both short- and long-term outcomes. Admission for resuscitated cardiac arrest, low perceived quality of life and ICU severity score correlated with in-hospital mortality; duration of mechanical ventilation and low previous functional abilities were associated with higher one-year mortality in hospital survivors.

## Supplementary Information

**Additional file 1.** Proxy’s questionnaire for patient’s initial assessment at ICU admission.

## Data Availability

The datasets used and/or analyzed during the current study are available from the corresponding author on reasonable request.

## Abbreviations

ADL: Activities of Daily Living
CIRS-G: Cumulative Illness Rating Scale for Geriatrics score
COPD: Chronic obstructive pulmonary disease
GDS: Geriatric Depression Scale
IADL: Instrumental Activities of Daily Living
ICU: Intensive care unit
SAPS II: Simplified Acute Physiology Score II
SOF: Study of Osteoporotic Fracture
SOFA: Sepsis-related Organ Failure Assessment

## References

[1] Ihra GC, Lehberger J, Hochrieser H, Bauer P, Schmutz R, Metnitz B (2012). Development of demographics and outcome of very old critically ill patients admitted to intensive care units. Intensive Care Med.

[2] Wunsch H, Guerra C, Barnato AE, Angus DC, Li G, Linde-Zwirble WT (2010). Three-year outcomes for Medicare beneficiaries who survive intensive care. JAMA.

[3] Lerolle N, Trinquart L, Bornstain C, Tadié J-M, Imbert A, Diehl J-L (2010). Increased intensity of treatment and decreased mortality in elderly patients in an intensive care unit over a decade. Crit Care Med.

[4] Ferrante LE, Pisani MA, Murphy TE, Gahbauer EA, Leo-Summers LS, Gill TM (2015). Functional trajectories among older persons before and after critical illness. JAMA Internal Med.

[5] Muscedere J, Waters B, Varambally A, Bagshaw SM, Boyd JG, Maslove D (2017). The impact of frailty on intensive care unit outcomes: a systematic review and meta-analysis. Intensive Care Med.

[6] Grimaldi D, Vincent J-L (2017). Clinical trial research in focus: rethinking trials in sepsis. Lancet Resp Med.

[7] Linn BS, Linn MW, Gurel L (1968). Cumulative illness rating scale. J Am Geriatr Soc.

[8] Ensrud KE, Ewing SK, Taylor BC, Fink HA, Cawthon PM, Stone KL (2008). Comparison of 2 frailty indexes for prediction of falls, disability, fractures, and death in older women. Arch Intern Med.

[9] Revel V, Haritchabalet I, Kervinio C, Drode M, Sauvanier M, Geny C, et al. Construction d’une échelle simplifiée pour la détection en médecine générale du fardeau de l’aidant d’une personne âgée dépendante. In: L’année gérontologique 2002 Vol 16 Tome 1. Paris: Serdi; 2002. p. 131–7.

[10] Katz S, Ford AB, Moskowitz RW, Jackson BA, Jaffe MW (1963). Studies of illness in the aged. The index of adl: a standardized measure of biological and psychosocial function. JAMA.

[11] Barberger-Gateau P, Commenges D, Gagnon M, Letenneur L, Sauvel C, Dartigues JF (1992). Instrumental activities of daily living as a screening tool for cognitive impairment and dementia in elderly community dwellers. J Am Geriatr Soc.

[12] Patrick DL, Danis M, Southerland LI, Hong G (1988). Quality of life following intensive care. J Gen Intern Med.

[13] Vincent JL, Moreno R, Takala J, Willatts S, De Mendonça A, Bruining H (1996). The SOFA (Sepsis-related Organ Failure Assessment) score to describe organ dysfunction/failure. On behalf of the Working Group on Sepsis-Related Problems of the European Society of Intensive Care Medicine. Intensive Care Med..

[14] Le Gall JR, Lemeshow S, Saulnier F (1993). A new Simplified Acute Physiology Score (SAPS II) based on a European/North American multicenter study. JAMA.

[15] Clément JP, Nassif RF, Léger JM, Marchan F (1997). Development and contribution to the validation of a brief French version of the Yesavage Geriatric Depression Scale. Encephale.

[16] Lipman RS (1982). Differentiating anxiety and depression in anxiety disorders: use of rating scales. Psychopharmacol Bull.

[17] Guralnik JM, Simonsick EM, Ferrucci L, Glynn RJ, Berkman LF, Blazer DG (1994). A short physical performance battery assessing lower extremity function: association with self-reported disability and prediction of mortality and nursing home admission. J Gerontol.

[18] Derouesne C, Poitreneau J, Hugonot L, Kalafat M, Dubois B, Laurent B. Mini-Mental State Examination: a useful method for the evaluation of the cognitive status of patients by the clinician. Consensual French version. Presse Med. 1999;28(21):1141–8. 10399508

[19] Likert, Rensis. A Technique for the Measurement of Attitudes. Archives of psychology. 1932;44–53.

[20] Raveau T, Annweiler C, Chudeau N, Gergaud S, Thiery S, Gautier J (2013). Comprehensive geriatric assessment in intensive care unit: a pilot study (pre-Seniorea). Geriatr Psychol Neuropsychiatr Vieil.

[21] Grambsch PM, Therneau TM (1994). Proportional hazards tests and diagnostics based on weighted residuals. Biometrika.

[22] Pietiläinen L, Hästbacka J, Bäcklund M, Parviainen I, Pettilä V, Reinikainen M (2018). Premorbid functional status as a predictor of 1-year mortality and functional status in intensive care patients aged 80 years or older. Intensive Care Med..

[23] Flaatten H, De Lange DW, Morandi A, Andersen FH, Artigas A, Bertolini G (2017). The impact of frailty on ICU and 30-day mortality and the level of care in very elderly patients (≥ 80 years). Intensive Care Med.

[24] Ferrante LE, Pisani MA, Murphy TE, Gahbauer EA, Leo-Summers LS, Gill TM (2016). Factors associated with functional recovery among older intensive care unit survivors. Am J Respir Crit Care Med..

[25] Heyland DK, Garland A, Bagshaw SM, Cook D, Rockwood K, Stelfox HT (2015). Recovery after critical illness in patients aged 80 years or older: a multi-center prospective observational cohort study. Intensive Care Med.

[26] for the VIP2 study group, Guidet B, de Lange DW, Boumendil A, Leaver S, Watson X, et al. The contribution of frailty, cognition, activity of daily life and comorbidities on outcome in acutely admitted patients over 80 years in European ICUs: the VIP2 study. Intensive Care Med. 2020;46(1):57–69. 10.1007/s00134-019-05853-1 PMC7223711 31784798

[27] Roch A, Wiramus S, Pauly V, Forel J-M, Guervilly C, Gainnier M (2011). Long-term outcome in medical patients aged 80 or over following admission to an intensive care unit. Crit Care.

[28] de Rooij SE, Govers A, Korevaar JC, Abu-Hanna A, Levi M, de Jonge E (2006). Short-term and long-term mortality in very elderly patients admitted to an intensive care unit. Intensive Care Med.

[29] Guidet B, Leblanc G, Simon T, Woimant M, Quenot J-P, Ganansia O (2017). Effect of systematic intensive care unit triage on long-term mortality among critically ill elderly patients in france: a randomized clinical trial. JAMA.

[30] Flaatten H, de Lange DW, Artigas A, Bin D, Moreno R, Christensen S (2017). The status of intensive care medicine research and a future agenda for very old patients in the ICU. Intensive Care Med.

[31] Kahneman D. Thinking, fast and slow. Toronto: Doubleday Canada; 2011 [cited 2020 Sep 6]. https://www.overdrive.com/search?q=20947F5B-F6A1-4CA7-B332-F46D587A0F8A

[32] Bowling A, Stenner P (2011). Which measure of quality of life performs best in older age? A comparison of the OPQOL, CASP-19 and WHOQOL-OLD. J Epidemiol Community Health.

