# Supplementary material for: Determinants of hospital and one-year mortality among older patients admitted to intensive care units: results from the multicentric SENIOREA cohort
Source: Ann Intensive Care. 2021 Feb 17;11:35. doi: 10.1186/s13613-021-00804-w (PMC7889762; doi:10.1186/s13613-021-00804-w)
Supplement: Supplementary file 1 — Additional file 1. Proxy’s questionnaire for patient’s initial assessment at ICU admission. [file 13613_2021_804_MOESM1_ESM.docx]

**Supplemental online materials**

**Proxy’s questionnaire for patient’s initial assessment at ICU admission.**

Questioning condition:

 On-site  Phone call

Respondent:

 Proxy  General practitioner  House caregiver

Cognitive assessment:

“ In your opinion, does your relative have memory disorders?”

 Yes  No

Quality of life:

“In you opinion, was your relative” :

 Verry happy  Happy  Unhappy  Very unhappy

“Is your relative energetic?”

 Yes  No  Don’t know

Pain

“In your opinion, is your relative painful?”

 No Pain  Mild pain  Moderate pain  Severe Pain

Anxiety

“In your opinion, is your relative anxious?”

 Yes  No

Depression

“In your opinion, is your relative depressed?”

 Yes  No
